# The relationship of different levels of high iodine and goiter in school children: a meta-analysis

**DOI:** 10.1186/s12986-021-00563-2

**Published:** 2021-05-03

**Authors:** Tingting Xu, Zhiyuan Ren, Shaohan Li, Long Tan, Wanqi Zhang

**Affiliations:** Department of Nutrition and Food Hygiene, School of Public Health, Tianjin Medical University, Heping District, No. 22, Qixiangtai Road, Tianjin, China

**Keywords:** High urinary iodine, High water iodine, Goiter, School children, Meta-analysis

## Abstract

**Background:**

Over the past decade, the phenomenon of high urine iodine (HUI) and high water iodine (HWI) has become more common. But the risk of goiter caused by different levels of HUI and HWI remains unclear.

**Objectives:**

To explore the risk of goiter development caused by HUI and HWI, and compare the risk of goiter development from different levels of high iodine.

**Methods:**

The Medline, Cochrane library, Embase, China National Knowledge Infrastructure and Wan fang databases were searched for relevant population-based studies investigating the link between high iodine levels and goiter development in mainland China. Three reviewers extracted data from the included studies independently, assessing the prevalence of goiter development due to high iodine.

**Results:**

Taking 100 μg/L ≤ UIC < 300 μg/L (UIC = urinary iodine concentration) as the reference group, the odds ratio (OR) regarding high iodine levels and goiter formation was 1.74 (95% CI 1.50, 2.01, *P* < 0.001), if the water iodine concentration (WIC) was greater than 100 μg/L, the OR between goiter development and WIC was 4.74 (95% CI 1.15, 19.46, *P* = 0.001). The Linear trend analysis of HUI and goiter showed that the prevalence of goiter increased with the increase of UIC (χ^2^ = 734.605, P < 0.001).

**Conclusions:**

When the UIC ≥ 300 μg/L or the WIC ≥ 100 μg/L, the risk of goiter will increase. The higher the UIC, the greater the risk of goiter development. In order to improve the public thyroid health, we should adhere to the monitoring of urinary iodine and water iodine, and keep them at an appropriate level.

**Trial registration:**

PROSPEROCR, CRD42020197620. Registered 8 August 2020, https://www.crd.york.ac.uk/PROSPERO/.

## Introduction

Iodine is an essential trace element in the human body and is a crucial component of both thyroxin and triiodothyronine, key hormones produced by the thyroid. Iodine exerts its physiological function through these hormones, promoting material and energy metabolism and allowing growth and development. Insufficient iodine intake can lead to an iodine deficiency, which in turn will manifest itself as specific health problems such as goiter development or endemic cretinism [1, 2]. The adopted global strategy of universal salt iodization has begun to combat iodine deficiencies and has significantly improved the health of the global population [3]. However, iodine intake must be kept in balance; excessive iodine levels will lead to adverse effects just as insufficient iodine levels will. An excessive iodine intake can lead to thyroid dysfunction; this can cause multiple health conditions including: hypothyroidism, hyperthyroidism, autoimmune thyroiditis and goiter formation [4, 5].

High iodine levels can be caused by dietary intake, with food high in iodine and iodized salt proving problematic; high background levels of iodine in drinking water may also cause this and exacerbate the problem of a high dietary intake. Both the WHO and the Chinese government recommended monitoring goiter rates in children as an indicator for long-term iodine nutrition in the population [6]. When iodine exposure is high, it is thought that the prevalence of children with goiters will exceed 5%. A cross-sectional survey of areas with high levels of iodine in the water was conducted in northern China; it was found that in area where water iodine levels exceeded than 300 μg/L, the population's urinary iodine concentration (UIC) was on average 476.3 μg/L, with a goiter prevalence of 10% [7]. It was also shown that when iodized table salt was consumed in areas with high iodine level water (iodine salt concentration of 10.4–34.1 mg/kg), the average UIC increased to 518 μg/L and the prevalence of goiters was 32.96% [8].

However, the risk of goiter development caused by different levels of high iodine intake was still unclear. Therefore, the aim of this study was to clarify the risk of high iodine intake on goiter development. It also aimed to evaluate the relationship between different high urinary iodine levels (HUI) and high water iodine (HWI) and the prevalence of goiters in these populations, using a meta-analysis approach.

## Materials and methods

The review was registered in the PROSPERO International Prospective Register of Systematic Reviews (https://www.crd.york.ac.uk/prospero; CRD number: 42020197620).

### Literature search

Medline, Embase, Cochrane library, China National Knowledge Infrastructure (CNKI) and Wan Fang databases were searched by computer from database inception to March 2020. The reference list generated was then manually searched. The Medline, Embase and Cochrane library database searches used the following search terms: (('Iodine' OR 'iodide') AND 'excess' OR 'high' OR 'excessive') AND 'goiter'. The search terms (“high or excess” and “iodine” and “goiter” (in Chinese)) were used to search the CNKI and Wan Fang databases.

### Inclusion and exclusion criteria

**Fig. 1:**
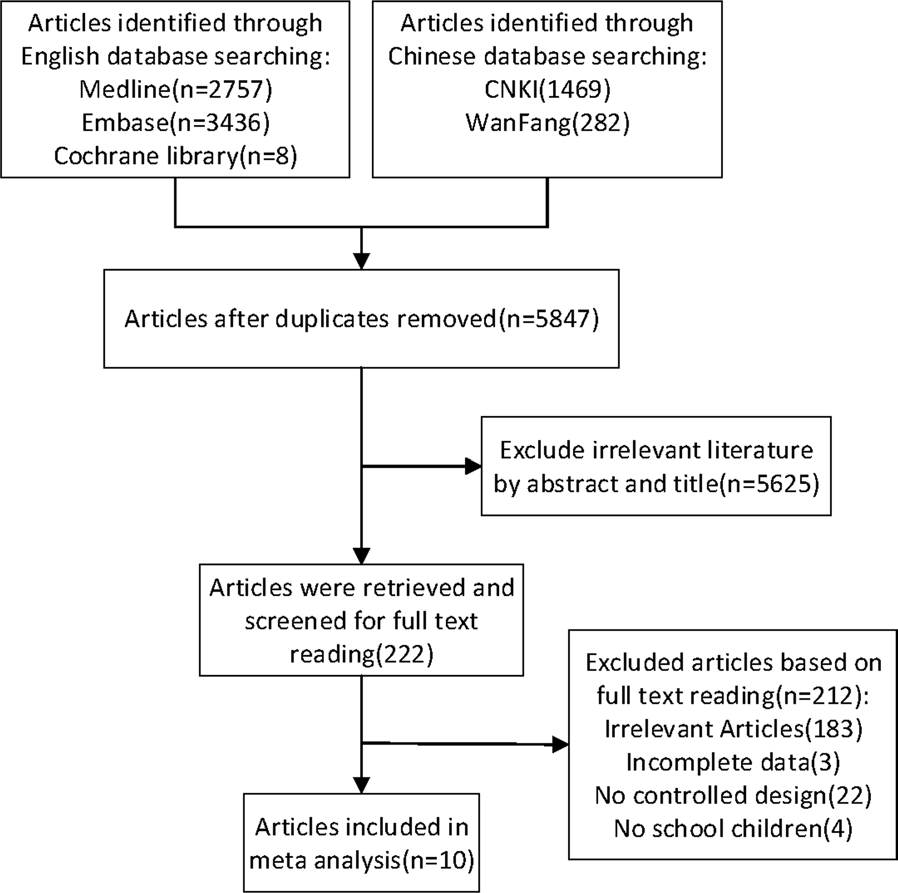
A flow chart of the literature search used for this meta-analysis

HUI level was defined in accordance with the standards of the WHO/UNICEF/ICCIDD [9] and HWI level was defined in accordance with the standards of GB19380-2016 [10]. The defined standard of HUI used 300 μg/L UIC as cut-off value for school-age children and adults; the standard for HWI level was defined as 100 μg /L water iodine concentration (WIC). Therefore, the following studies were included in this meta-analysis (Fig. 1): (1) cross-sectional studies that defined a high iodine intake group using a UIC cut-off of 300 μg/L for school-age children, with controls selected from populations with average or slightly elevated intakes (cut-off values for average and slightly elevated iodine intakes were 100–200 μg/L and 200–300 μg/L UIC in school-age children, respectively); (2) cross-sectional studies that defined the group of areas with high water iodine levels using a WIC cut-off of 100 μg/L, whereas the controls were selected from populations with normal water iodine levels ( Normal water iodine levels were ≤ 100 μg/L in WIC). In cases of repeated publication, only the first published article was included. Only articles published in English or Chinese were considered. Animal studies, case reports, reviews, studies not reporting sample size and studies with incomplete data, were all excluded.

### Data extraction and quality evaluation

Data was independently extracted by three authors from the included studies (Li SH, Ren ZY and Xu TT). When the results from the literature were uncertain, it was discussed between the authors before reaching a final agreement. The following data were extracted from included literatures: surname of first author, publish time, study time, UIC, WIC, the number of patients with goiters, total number of researchers and the prevalence of goiters. The methodological quality of the included studies was assessed using the cross-sectional study evaluation scale recommended by the Agency for Healthcare Research and Quality (AHRQ) [11]. There were 11 criteria to this scale. Each criterion included three options: “yes”, “no” and “unclear”. A “yes” scored 1 point whereas “unclear” or “no” scored 0 points. Article quality was assessed as follows: low quality = 0–3; moderate quality = 4–7; high quality = 8–11. The evaluation results of the 11 studies included in the meta-analysis are shown in Table 1. This assessment was performed by 2 authors (Li SH and Ren ZY) independently, with a third author (Xu TT) being consulted to settle disagreements.

**Table 1:** The characteristics of 14 cross-sectional studies

Author	Publish time	Study time	UIC (μg/L)	WIC (mg/L)	n	N	Prevalence of goiter	Quality evaluation
Liu [15]	2010	1999	13.2	–	12	82	14.6	7
			37.3	–	20	379	5.2	
			77.7	–	89	1015	8.8	
			125.5	–	84	1193	7.0	
			174.3	–	78	1329	5.9	
			248.1	–	199	2731	7.3	
			378.0	–	350	3682	9.5	
			599.1	–	237	2026	11.7	
			865.3	–	44	364	12.2	
			1157.5	–	14	183	7.5	
		2002	13.6	–	14	108	13.0	
			36.2	–	14	297	4.7	
			77.3	–	47	876	5.4	
			125.7	–	48	1225	3.9	
			174.7	–	57	1321	4.3	
			245.2	–	135	2322	5.8	
			371.0	–	140	2160	6.5	
			547.7	–	48	650	7.4	
			862.2	–	11	86	12.9	
			1274.4	–	3	56	6.0	
		2005	10.6	–	19	172	11.0	
			36.0	–	33	426	7.7	
			77.7	–	62	1109	5.6	
			125.9	–	64	1392	4.6	
			175.8	–	72	1503	4.8	
			251.8	–	130	3012	4.3	
			368.1	–	141	2476	5.7	
			574.8	–	39	703	5.5	
			868.0	–	2	77	2.6	
			1200.0	–	7	69	10.1	
		2005	11.3	–	1	53	1.9	
			37.2	–	5	126	4.0	
			76.5	–	22	386	5.7	
			127.5	–	39	662	5.9	
			175.2	–	47	783	6.0	
			252.2	–	142	1824	7.8	
			393.8	–	452	3895	11.6	
			762.0	–	359	2621	13.7	
			868.0	–	114	675	16.9	
			1200.0	–	273	1531	17.8	
		2007	13.2	–	237	2419	9.8	
			46.2	–	807	6612	12.2	
			81.0	–	786	9251	8.5	
			124.4	–	548	7205	7.6	
			154.6	–	454	6401	7.1	
			244.5	–	832	10,274	8.1	
			357.2	–	764	8129	9.4	
			575.4	–	195	1623	12.0	
			875.6	–	11	96	11.5	
			1273.4	–	13	77	16.9	
Wang [16]	2015	2012	271.0	–	11	291	3.8	7
			692.6	–	28	300	9.3	
Xiao [17]	2011	2007	319.2	–	61	731	8.3	7
			189.8	–	69	1248	5.5	
Jia [18]	2014	2014	74.3	73.8	11	196	5.6	7
			312.8	144.7	25	189	13.2	
			455.6	258.5	20	158	12.6	
			793.5	501.0	18	165	10.9	
Wang [19]	2015	2014	514.0	304.4	9	100	9.0	7
			196.17	141.6	1	100	1.0	
Yu [20]	2008	1999	83.5	–	192	2708	7.1	10
			242.9	–	119	2708	4.4	
			650.9	–	187	2708	6.9	
Liu [21]	2007	–	480.4	> 150.0	108	1458	7.4	6
			228.0	≤ 150.0	22	1229	1.8	
Tang [22]	2006	–	612.5	124.2 ± 88.2	141	1184	11.9	6
			269.4	136.6 ± 89.2	95	889	10.7	
			642.9	183.0 ± 190.2	148	1133	13.1	6
			244.5	124.1 ± 105.1	129	862	9.7	
			499.7	112.3 ± 85.3	114	1032	11.1	
Jia [23]	2006	–	460.5	> 150.0	84	570	14.7	
					35	269	13.1	
			310.3	≤ 150.0	60	656	9.1	6
					23	316	7.3	
Dai [24]	2013	2004–2010	433.3	–	55	211	26.1	
			296.1	–	16	235	6.8	
			313.9	–	20	216	9.3	7
			345.9	–	19	208	9.1	
			199.8	–	14	237	5.9	
			–	≤ 100.0	91	247	36.8	
			–	100.0–200.0	573	2019	26.4	
			–	200.0–300.0	294	1189	24.7	
			–	300.0–400.0	205	648	31.6	
			–	> 400.0	741	2230	33.2	

### Statistical analysis

Stata software 16.0 and RevMan 5.3 were used to perform the meta-analysis. The odds ratio (OR) and 95% confidence interval (CI) were the statistical effect size used to estimate the effect of iodine exposure. A *P* < 0.05 was considered statistically significant. The I^2^ test was used to quantify heterogeneity [12]. According to the Cochrane Handbook for Systematic Reviews [13], if the I^2^ value was less than 50%, the heterogeneity could be accepted, and the fixed-effects model was used. If high levels of heterogeneity (I^2^ > 50%) were detected between the studies, the random-effects model was selected. If different subgroups of the same group required different models, the random-effects model was used. The presence of publication bias was examined using the Begg’s test [14], *P* > 0.1 was considered statistically significant. The subgroup analyses of the prevalence of goiter with different HUI and the prevalence of goiter with different HWI would be done. Mantel Haenszel χ^2^ test was used for linear trend analysis of HI and goiter.

## Results

### Literature search and the characteristics of the included studies

During the initial database search, 5847 articles were retrieved. Following an initial screening based on the paper title and abstract, 222 articles were retrieved and screened for full text reading. Of the 222 papers, 183 were found irrelevant, 3 had incomplete data, 22 did not have a controlled design and 4 did not examine school age children. Consequently, 10 studies were included in the meta-analysis [15–24] (Fig. 1). The characteristics of these studies are listed in Table 1.

### High urinary iodine and goiter

**Fig. 2:**
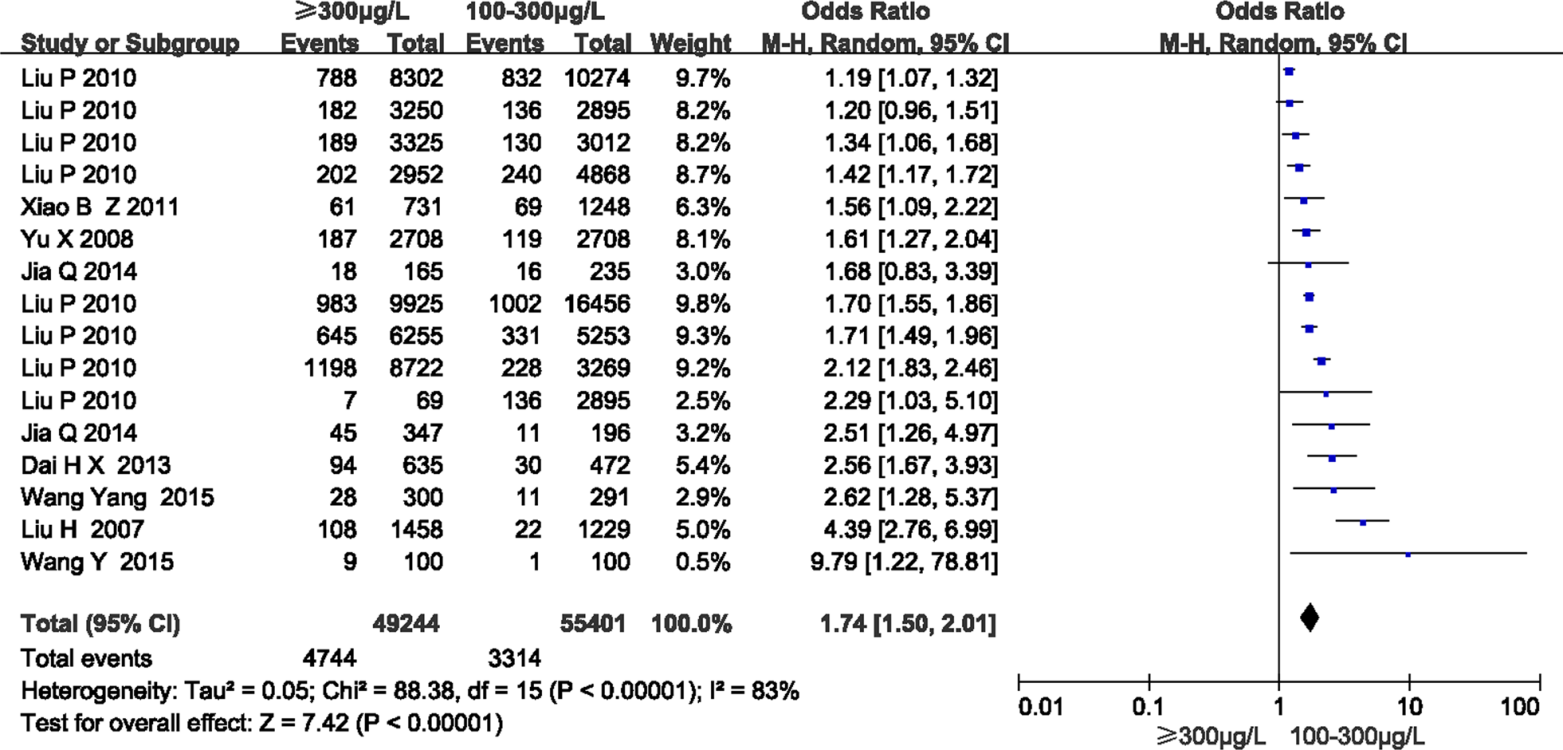
A forest plot of results for a UIC ≥ 300 μg/L and the prevalence of goiters

The association between HUI and the prevalence of goiters is shown in Fig. 2. A UIC of 100–300 μg/L was selected as the reference group. 7 articles reported the prevalence of goiters, which included 16 studies. These 16 studies included 104,645 subjects. There were 49,244 subjects from the UIC ≥ 300 μg/L group and 55,401 subjects from the 100–300 μg/L group. The OR value was 1.74 (95% CI 1.50, 2.01, *P* < 0.001). It indicated that a UIC ≥ 300 μg/L was associated with an increased risk of developing a goiter compared to a UIC from 100–300 μg/L. The result of the Begg’s test and Egger’s test were *P* = 0.163 > 0.1, indicating that there was no significant publication bias.

### High water iodine and goiter

**Fig. 3:**
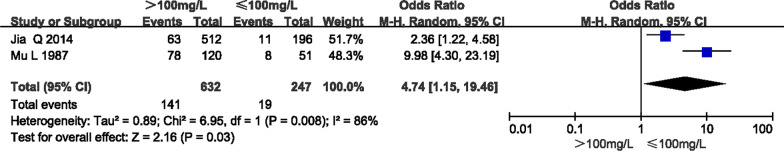
Forest plot of result of WIC ≥ 100 μg/L and the prevalence of goiters

The association between HWI (> 100 μg/L) and the development of goiters was shown in Fig. 3. Three articles reported the development of goiters. These studies included 879 subjects (632 subjects from the high iodine group and 247 subjects from the control group). None of the studies crossed the invalid line. The OR value was 4.74 (95% CI 1.15, 19.46, *P* = 0.008). This indicated that a WIC > 100 μg/L was associated with an increased risk of goiter development compared with WI ≤ 100 μg/L. Owing to the limited number of studies included in this aspect of the analysis, publication bias was not assessed.

### Subgroup analysis

#### Prevalence of goiter with different high urinary iodine levels

**Fig. 4:**
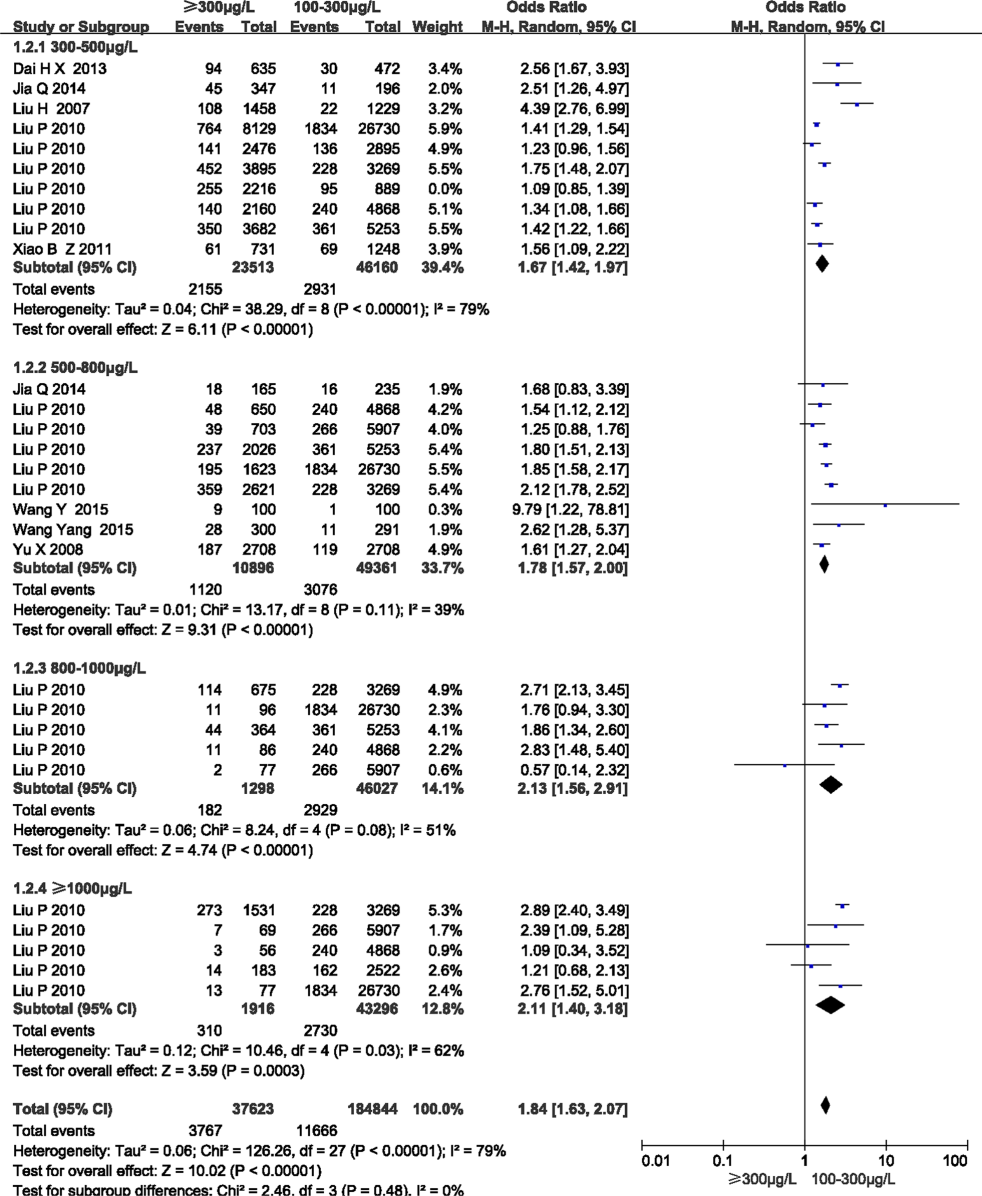
Forest plot of subgroup analysis of results of a UIC ≥ 300 μg/L and the prevalence of goiters

The associations between different levels of HUI and the prevalence of goiters were shown in Fig. 4. Taking a UIC of 100–300 μg/L as the reference group, the risk of goiter development was assessed in groups with a UIC of 300–500 μg/L, 500–800 μg/L, 800–1000 μg/L and ≥ 1000 μg/L. The OR values were 1.67 (95% CI 1.42, 1.97, *P* < 0.001), 1.78 (95% CI 1.57, 2.00, *P* < 0.001), 2.13 (95% CI 1.56, 2.91, *P* < 0.001) and 2.11 (95% CI 1.40, 3.18, *P* < 0.001), respectively. The overall risk of high iodine causing goiter development was OR = 1.84 (95% CI 1.63, 2.07, *P* < 0.001).

### Linear trend analysis of high iodine and goiter

#### Linear trend analysis of HUI and goiter

The linear-by-linear association between different levels of HUI and the prevalence of goiter are shown in Table 2. The urine iodine concentration was divided into five groups from low to high as follows: 100–300, 300–500, 500–800, 800–1000, ≥ 1000μg/L for Mantel Haenszel χ^2^ test. The result showed that there was a linear correlation between the UIC and the prevalence of goiter (χ^2^ = 734.605, P < 0.001). And with the increase of UIC, the prevalence of goiter also increased.

**Table 2:** mantel Haenszel χ^2^ test of UIC and goiter

UIC (μg/l)	Goiter	Not goiter	χ^2^	*P*
100–300	3405	53,918	734.605	< 0.001
300–500	2499	24,157		
500–800	1381	11,732		
800––1000	182	1116		
≥ 1000	310	1606		

## Discussion

This study explored the relationship between high iodine levels and the prevalence of goiter development through a meta-analysis of the 10 cross-sectional studies, using UIC and WIC as indicators of iodine exposure. For ethical reasons, it is difficult to conduct a randomized controlled trial exploring the effects of high iodine exposure on goiter development. Therefore, it was speculated that the inclusion of cross-sectional studies in our meta-analysis might help to account for the association between high iodine levels and goiter development among school children.

UIC was the most common and practical marker used to estimate population iodine levels and their iodine intake [25, 26]. This is because > 90% of dietary iodine is readily excreted in urine [27]. Using urinary iodine levels as a marker for iodine exposure, it was found that there was a higher prevalence of goiter development in people with a high iodine intake (UIC ≥ 300 μg/L) when compared to those with a normal and slightly elevated intake (100 μg/L ≤ UIC < 300 μg/L). Moreover, the linear trend analysis showed that the prevalence of goiter development generally increased as iodine intake increased.

Water is a key resource which people ingest every day, if the concentration of iodine in the water is too high, it will affect the levels of iodine in the human body. After 2016, a WIC > 100 μg/L was defined as high level of iodine in water (GB/T 19380-2016). In this meta-analysis, it was found that when the WI was greater than 100 mg/L, the OR of goiter development was 4.47 (95% CI 1.15, 19.46, *P* < 0.001). Though the results of the Jia Q Z, and Tang Z C’s study cross the invalid line and lack statistical significance, the OR values of the remaining studies within the meta-analysis were greater than 1; this suggests that high iodine levels lead to an increased risk of goiter development.

A Goiter is the enlargement of the thyroid. There was research found that excessive iodine intake will increase the synthesis of thyroid hormones and change the antigenicity of thyroglobulin, leading to the accumulation of colloid in the thyroid follicular cavity and flattening of thyroid follicular epithelial cells [28]. This is a typical pathological manifestation of goiter caused by high iodine. The decrease in thyroid hormone production caused by high iodine levels is called the Wolff-Chaik off effect [29]. Normally, thyroid hormone levels return to normal after a few days; this is termed the so-called “escape” phenomenon [30]. Although the mechanism by which high iodine causes goiter development remains unclear, failure to “escape” is considered to play a role. In addition, the continuous stimulation of thyroid stimulating antibodies activate NIS and/or spread lymphocyte infiltration; this may also play a role in high iodine levels causing the development of a goiter [31].

Clarifying the risk of high iodine on goiter development has important clinical and public health significance. Iodine is the main raw material in the synthesis of thyroxin. Changes in environmental iodine levels directly affect iodine intake; this in turn affects the subsequent changes in the synthesis and secretion of thyroid hormones. A study explored the effect of high parental iodine levels on the thyroid hormone levels of offspring. First, parental mice were dosed with iodine using tap water containing iodine (3000 μg/L) for 4 months. At the end of the fourth month, the mice were paired for mating. The levels of thyroid hormone and TSH in the serum and brain of the offspring mice were then measured after birth. It was found that on the 14th day post-birth, the serum T4 level in the high iodine group was significantly reduced, whilst the serum TSH was higher than that of the control group [32]. Most healthy individuals can tolerate a high iodine intake. However, in certain susceptible populations, an excessive iodine intake may lead to hyperthyroidism [33], hypothyroidism [34], thyroid enlargement [35] and thyroid autoimmunity [36].

Considering the difficulty of conducting randomized trials and the absence of a meta-analysis of the relationship between high iodine intake and the development of a goiter, this study is of great significance for public health managers. These results confirm the necessity of monitoring iodine concentration in water and urine. The development of a goiter is a sensitive marker that reflects the long-term impact of a high iodine intake effect; the prevalence of goiters within a population has traditionally been a marker that reflects the long-term iodine status of a population.

This meta-analysis had several advantages. Firstly, this was the first meta-analysis assessing both UIC and WIC as iodine exposure markers; it was also the first which explored the risk of different high iodine levels on goiter development. secondly, it further confirmed that HUI and HWI can cause goiters, whilst also showing that urinary iodine and water iodine levels can be used as indicators of iodine exposure. Thirdly, in order to ensure the research quality of this meta-analysis, strict inclusion criteria were developed which helped to increase the robustness of the data. Finally, an extensive article retrieval and data extraction was conducted by 3 independent reviewers. At the same time, there are several limitations to the review. The included studies were cross-sectional studies, so the study is unable to determine causality between high iodine intake and goiter development. Also, as there is only limited relevant literatures for newborns, pregnant women, the elderly and other groups, only included school-age children were investigated in this meta-analysis. Further researches needed to be done to explore the relationship between high iodine levels and goiter development in newborns, pregnant women and the elderly.

## Conclusions

In conclusion, the results of this meta-analysis showed that HUI or HWI increase the risk of the development of goiters. It also showed that as concentration of urinary iodine or water increased; the risk of goiter increaed.

## Data Availability

The datasets used and/or analysed during the current study are available from the corresponding author on reasonable request.

## Abbreviations

USI: Universal salt iodization
HUI: High urinary iodine
HWI: High water iodine
CNKI: China National Knowledge Infrastructure
UIC: Urinary Iodine concentration
WIC: Water iodine concentration
OR: Odds ratio
CI: Confidence interval
